# Premature Dentition

**Published:** 1839-08

**Authors:** Solyman Brown


### PREMATURE DENTITION.

[ BY SOLYMAN BROWN. ]

As grave historians have thought it a fact worthy of record, that Louis XIV was born with a tooth manifesting itself through the gums, which was esteemed by many as a token of his future greatness, and as it is now known that such an occurrence is far from being either wonderful or miraculous, I shall give an instance which has come within my personal knowledge, of a lady among my patients, who has had three daughters born with two teeth each.

The mother, who is a highly respected and intelligent lady of this city, lately presented me with one of these teeth, which was fully developed at birth ; and which was extracted under the popular belief that such teeth incommode the mother in lactation. Subsequently, two others of her children had at birth a similar developement of the lower central incisors, which were suffered to remain through the entire period of lactation without the slightest inconvenience to the maternal nurse. These facts may serve to regulate dental practice in similar circumstances by teaching that such teeth may be suffered to remain with perfect safety in all cases where they occasion no inconvenience.

